# Harmonization of Cognitive Data Across Cohort Studies

**DOI:** 10.1093/geroni/igaf122.577

**Published:** 2025-12-31

**Authors:** Alden Gross, Adam Spira, Emerson M Wickwire, Jennifer Albrecht, Atul Malhotra, Halima Amjad, Marcela Blinka, Christopher Kaufmann

**Affiliations:** Johns Hopkins Bloomberg School of Public Health, Baltimore, Maryland, United States; Johns Hopkins Bloomberg School of Public Health, Baltimore, Maryland, United States; University of Maryland, Baltimore, Maryland, United States; University of Maryland, Baltimore, Maryland, United States; University of California, San Diego, San Diego, California, United States; Johns Hopkins University School of Medicine, Baltimore, Maryland, United States; Johns Hopkins University, Center on Aging and Health, Baltimore, Maryland, United States; University of Florida, Gainesville, Florida, United States

## Abstract

Harmonizing longitudinal population-based studies affords the ability to compare cognitive decline even when protocols for measuring cognition differ between cohorts. We derived a longitudinally harmonized general cognitive factor score based on cognitive tests from the Health and Retirement Study (HRS) and the National Health and Aging Trends Study (NHATS), two of the largest population-based studies with longitudinal cognitive testing in the US. Using item response theory methods supplemented by an item banking approach, we leveraged common and unique tests administered in 2012-2018 Core interviews of the two studies to derive a harmonized general cognitive factor score. We evaluated concurrent and predictive criterion validity of harmonized scores by: 1) cross-sectionally comparing scores at baseline (2012) with cognitive impairment status and demographics; and 2) longitudinally assessing associations of demographics and risk factors. Cross-sectionally, the harmonized score was lowest among dementia/probable dementia groups in both HRS (z=-1.55 SD) and in NHATS (z=-2.21 SD) compared to cognitively normal participants in HRS (z = 0.27 SD) and NHATS (z=-0.05 SD). Longitudinally, in both studies, cognitive decline is steeper for participants at older ages within participants, with less education, and those reporting hypertension, stroke, or diabetes, relative to those without. This harmonized cognitive score can be a resource for aging researchers to examine and compare rare risk factors for cognitive performance and decline across large population-based studies. It returns patterns of change across demographic and health groups consistent with prior literature.

